# A review of subtidal kelp forests in Ireland: From first descriptions to new habitat monitoring techniques

**DOI:** 10.1002/ece3.6345

**Published:** 2020-06-16

**Authors:** Kathryn M. Schoenrock, Kenan M. Chan, Tony O'Callaghan, Rory O'Callaghan, Aaron Golden, Stacy A. Krueger‐Hadfield, Anne Marie Power

**Affiliations:** ^1^ Department of Zoology School of Natural Sciences Ryan Institute NUI Galway Galway Ireland; ^2^ Seasearch Ireland Comhairle Fo‐Thuinn Galway Ireland; ^3^ Áras de Brún School of Mathematics, Statistics and Applied Mathematics NUI Galway Galway Ireland; ^4^ Department of Biology CAS University of Alabama Birmingham AL USA

**Keywords:** indicator species, *Laminaria hyperborea*, long‐term ecological research, marine ecosystems, monitoring, remote sensing, resilience

## Abstract

**Aim:**

Kelp forests worldwide are important marine ecosystems that foster high primary to secondary productivity and multiple ecosystem services. These ecosystems are increasingly under threat from extreme storms, changing ocean temperatures, harvesting, and greater herbivore pressure at regional and global scales, necessitating urgent documentation of their historical to present‐day distributions. Species range shifts to higher latitudes have already been documented in some species that dominate subtidal habitats within Europe. Very little is known about kelp forest ecosystems in Ireland, where rocky coastlines are dominated by *Laminaria hyperborea*. In order to rectify this substantial knowledge gap, we compiled historical records from an array of sources to present historical distribution, kelp and kelp forest recording effort over time, and present rational for the monitoring of kelp habitats to better understand ecosystem resilience.

**Location:**

Ireland (Northern Ireland and Éire).

**Methods:**

Herbaria, literature from the Linnaean society dating back to late 1700s, journal articles, government reports, and online databases were scoured for information on *L. hyperborea*. Information about kelp ecosystems was solicited from dive clubs and citizen science groups that are active along Ireland's coastlines.

**Results:**

Data were used to create distribution maps and analyze methodology and technology used to record *L. hyperborea* presence and kelp ecosystems within Ireland. We discuss the recent surge in studies on Irish kelp ecosystems, fauna associated with kelp ecosystems that may be used as indicators of ecosystem health and suggest methodologies for continued monitoring.

**Main Conclusions:**

While there has been a steady increase in recording effort of the dominant subtidal kelp forest species, *L. hyperborea*, only recently have studies begun to address other important eco‐evolutionary processes at work in kelp forests including connectivity among kelp populations in Ireland. Further monitoring, using suggested methodologies, is required to better understand the resilience of kelp ecosystems in Ireland.

## Graphical abstract

*Laminaria hyperborea* is a marine ecosystem engineer that structures sub‐tidal kelp forests in Europe. In Ireland this species has been recorded since 1913, but kelp ecosystem function and resilience is not well understood. This review provides analysis of kelp records and the few ecosystem studies that exist to suggest future areas of research to better understand and protect these natural resources.

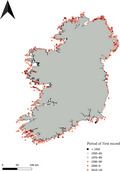

## 1 BACKGROUND

Large subtidal brown algal species form extensive underwater forests along the Irish coastline. Dominant species include the orders Fucales (*Himanthalia elongata* (Linnaeus) S.F.Gray 1821 and *Cystoseira* spp.), Tilopteridales (*Sacchoriza polyschides* (Lightfoot) Batters), and Laminariales (*Laminaria digitata* (Hudson) J.V.Lamouroux, *Alaria esculenta* (Linnaeus) Greville, *Saccharina latissima* (Linnaeus) C.E.Lane, C.Mayes, Druehl & G.W.Saunders, and *L. hyperborea* (Gunnerus) Foslie). Recently, non‐native species have arrived in these subtidal ecosystems via poleward distribution shifts (e.g., Laminariales—*L. ochroleuca* Bachelot de la Pylaie; Schoenrock, O'Callaghan, O'Callaghan, & Krueger‐Hadfield, 2019) or from aquaculture and/or shipping practices (Fucales—*Sargassum muticum* (Yendo) Fensholt 1955; Laminariales—*Undaria pinnatifida* (Harvey) Suringar; Kraan, 2017). Along semi‐exposed rocky coastlines, *L. hyperborea* forms dense forests, whereas in the calmer, shallow regions of tidal loughs and fjords, *S. latissima* can form small forests on hard substratum. The paucity of subtidal research makes it difficult to determine the current and historical scope of these habitats along Ireland's shorelines, but the synergistic threats of ocean warming and increased commercial harvesting present a critical need to better understand the historical and present‐day distribution of these important species in order to protect current and future marine forests.

As ecosystem engineers (Jones, Lawton, & Shachak, 1994), kelp provides structure for shallow marine habitats as a resource and a habitat for many organisms (>300 macrofauna species in *L. hyperborea* forests in Ireland throughout the year (Schoenrock et al., 2020). In other parts of Europe, kelp forests are foraging habitats for marine birds and fish (Norderhaug, Christie, Fossa, & Frederiksen, 2005; Norderhaug, Fredriksen, & Nygaard, 2003), provide substrata for diverse marine assemblages (Bengtsson, Sjøtun, Lanzén, & Øvreås, 2012; Christie, Jørgensen, & Norderhaug, 2007; Christie, Jörgensen, Norderhaug, & Waage‐Nielsen, 2003; Norderhaug, Christie, & Rinde, 2002; Schoenrock et al., 2020), and are the basis of multiple food chains from coastal habitats to the deep sea (Filbee‐Dexter, Wernberg, Norehaug, Ramirez‐Llodra, & Pedersen, 2018), even providing a carbon resource for phytoplankton (Fredriksen, 2003). These habitats can also dampen wave energy, protecting coastlines from erosion (Lovas & Tørum, 2001), and modify parameters of the marine environment, including carbonate chemistry and light attenuation (Dean, 1985; Hofmann et al., 2011; Krause‐Jensen et al., 2015). In Ireland, observations indicate kelp forests are seasonal homes to commercial species like the edible crab (*Cancer pagurus* Linnaeus), European lobster (*Homarus gammarus* Linnaeus), and multiple species of juvenile fish that inhabit the kelp canopy (Schoenrock et al., 2020). There are natural seasonal patterns in these marine communities, but certain species inhabit kelp forests throughout the year including echinoderms, such as the common star (<3 cm in diameter, *Asterias rubens* Linnaeus), spiny sea star (*Marthasterias glacialis* Jullien), and the common urchin (*Echinus esculentus* Linnaeus); a full summary of species is provided in Table 1, (Schoenrock et al., 2020). The constant presence of these taxa in kelp forests over two years of monitoring suggests that they are indicators of healthy ecosystems in the west of Ireland. Regions to the north, south, and east have similar communities (Schoenrock et al., 2020), but these regions have not been as thoroughly surveyed as the west of Ireland.

**TABLE 1:** A list of “indicator species” that are consistently associated with healthy *Laminaria hyperborea* forests throughout the year, including their average abundance per m^−2^ of kelp forest and trophic function (Schoenrock et al., 2020)

Species	Average abundance m^−2^	Trophic function
Sponge, encrusting *Suberites* spp.	0.26	Filter feeder
Hydroid, *Obelia geniculata*	0.27	Filter feeder (on kelp)
Anemone, *Urticina feline*	1.54	Predator
Cnidarian, *Caryophyllia smithii*	0.59	Filter feeder
Annelid, *Eupolymnia nebulosa*	0.02	Filter feeder
Crustacean, *Palaemon serratus*	0.016	Predator
Mollusc, *Gibbula umbilicalis*	0.86	Grazer
Mollusc, *Gibbula cineraria*	0.06	Grazer
Echinoderm, *Asterias rubens* (<3 cm diameter)	1.22	Predator
Echinoderm, *Marthasterias glacialis*	0.41	Predator
Echinoderm, *Holothuria forskali*	0.39	Suspension feeder
Echinoderm, *Echinus esculentus*	0.2	Grazer
Ascidian, *Aplidium punctum*	0.78	Filter feeder
Ascidian, *Distomus variolosus*	4.99	Filter feeder
Ascidian, *Diplostoma spongiforme*	0.08	Filter feeder
Vertebrate, *Pomatoschistus* spp.	0.015	Predator
Vertebrate, *Gobiusculus flavescens*	0.39	Predator

Note

A lack of these species within a *L. hyperborea* forest may indicate (a) the ecosystem is unhealthy or (b) the habitat is small (“kelp park” see Parr, 2020) or comprised of mixed kelps (see Table 5 where communities change with kelp species).

Kelp forest decline has been observed world‐wide driven by warming oceans and heatwave events, anthropogenic inputs (harvesting and eutrophication), and herbivore pressure (Krumhansl et al., 2016; Reed et al., 2016; Wernberg, Krumhansl, Filbee‐Dexter, & Pedersen, 2019). In Europe, the distribution of kelp species has changed over time with climate forcing (from the last glacial maximum), and species are predicted to continue retracting their southern range and move northward with ocean warming (Assis, Araújo, Araújo, & Serrão, 2018; Assis, Araújo, et al., 2018; Assis, Lucas, Bárbara, & Serrão, 2016; Assis, Serrão, Coelho, Tempera, et al., 2018). Currently warm‐water, subtidal forests dominated by *L. ochroleuca* have retracted from their southern range edges in Morocco to current limits mid‐Portugal (Assis, Araújo, et al., 2018; Assis, Serrão, Coelho, Tempera, et al., 2018), while the cold‐water kelp *L. hyperborea* has retracted its southern range edge from the Portuguese coastline to the Spanish coastline of the Bay of Biscay (Assis, Araújo, et al., 2018). Marine forests from the Mediterranean coast to southern Portugal are poorly understood, though it seems many species ranges are retracting at their lower latitude range limits. On the other hand, at higher latitudes, abundances of some species, such as *S. latissima* and *S. polyschides*, are increasing at their northern range edges, while the spread of invasive species, such as *U. pinnatifida,* is increasing (Araujo et al., 2016). In addition, herbivore pressure is devastating *L. hyperborea* at its northern ranges (northern Norway), though urchin populations can fluctuate from year to year, allowing regrowth of some populations (Hagen, 1995). Harvesting is also a threat to kelp forests across Europe, particularly France and Norway where the commercial exploitation of *L. hyperborea* and *L. digitata* has been occurring for decades (Valero, Engel, Billot, Kloareg, & Destombe, 2001). The mechanical removal of *L. hyperborea* results in the removal of whole individuals. Recovery can take greater than 5 years (Lorentsen, Sjøtun, & Grémillet, 2010), and populations in the heaviest areas of harvest may be important reservoirs of genetic diversity (e.g., Brittany, France: Robuchon, Couceiro, Peters, Destombe, & Valero, 2014).

Kelp have been of great interest to industry and science in the past, in flux with economic, socio‐political, and technical advances beginning during the 1700s. Therefore, there are many historical accounts of seaweed from sea captains, fisheries, and naturalists along the Irish coastline from the late 1700s to the present day. These qualitative historical records provide perspective on the value of kelp along Ireland's coastline, but given the pressures outlined above, we need a better understanding and documentation of kelp ecosystems. In this review, we focus on the presence of the subtidal species *L. hyperborea* from 1700s Ireland to present day, collating disparate historical records for the first time. This review significantly contributes to our understanding of kelp forest function in Ireland at a time when interest in kelp harvesting is increasing despite the fact we do not understand basic ecological and evolutionary processes at work in these systems. We show that regular and systematic monitoring is urgently needed in order to conserve and inform policy makers to foster resilience, which we define as the ability of this ecosystem to recover from a disturbance and maintain ecosystem function.

## 2 *LAMINARIA HYPERBOREA* RECORDS IN IRELAND

**FIGURE 1:**
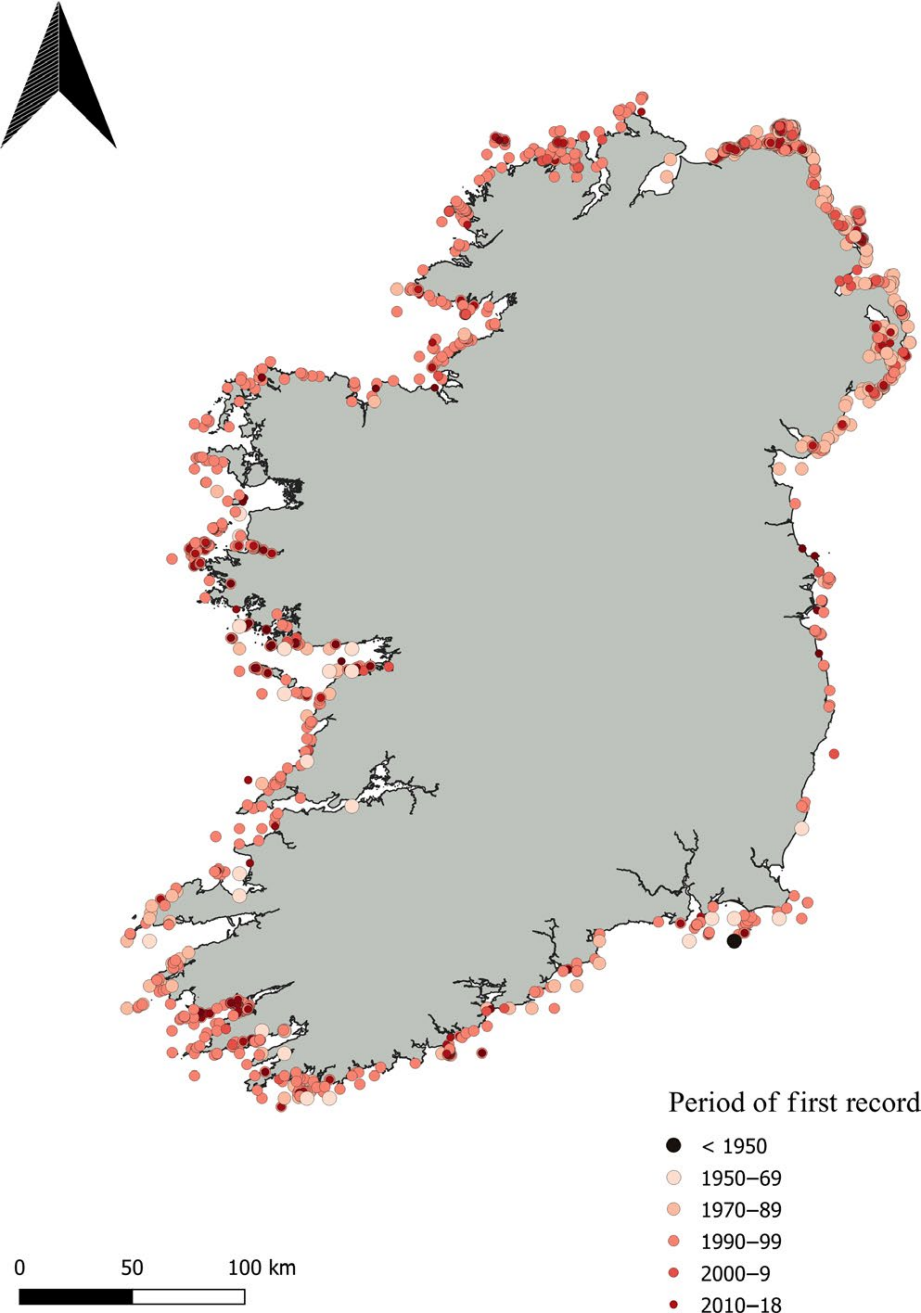
*Laminaria hyperborea* “period of first record” for Ireland from pre‐1950 (1913 was the only record), from 1950–1969, 1970–1989, 1990–1999, 2000–2009, and 2010 to 2018 (most recent year of record on data platforms)

**FIGURE 2:**
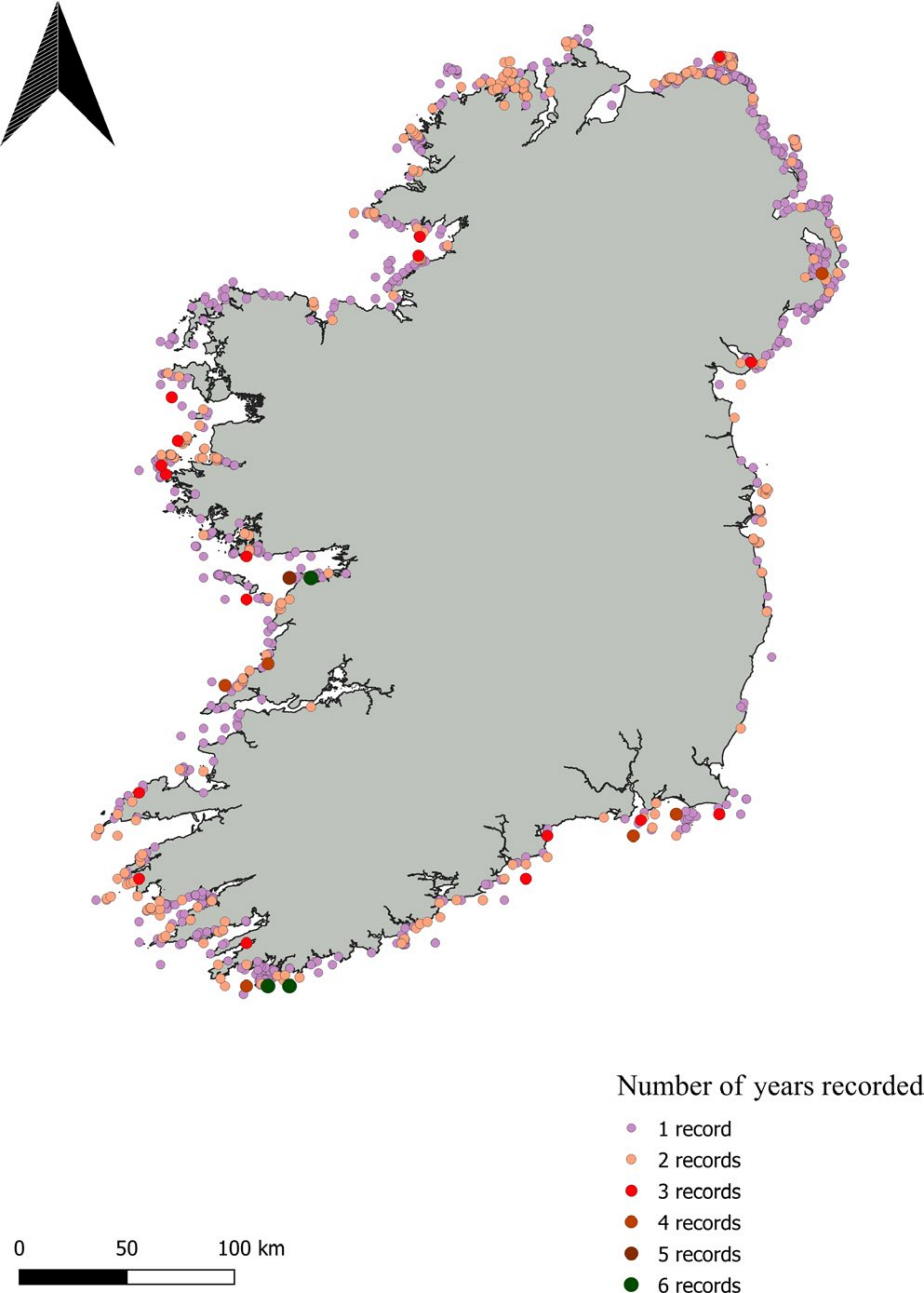
Recording effort, “number of years recorded”,of *Laminaria hyperborea* along the coast of Ireland from 1913 to present day

In November 2019, the written records of phycologists and old texts were accessed in the Linnaean Society of London to investigate the study of *L. hyperborea* from 1700 to present day. National herbaria were visited to examine kelp voucher specimens from Ireland over the same time period, though we must note that there are, in general, very few records of large brown algae in these herbaria: National University of Ireland Galway (M. Guiry, 1 record, Finavarra County Clare), The National Botanic Gardens of Ireland (2 records, Clifden County Galway), Trinity College (0 records), and Natural History Museum of London (3 records, Clare Island). The lack of voucher specimens is likely due to the difficulty in preserving thick thalli on paper, and many specimens were likely placed in formalin rather than pressed for preservation (Tsuda & Abbott, 1985). Precise collection details were not noted on many herbarium sheets making it difficult to ascertain the location of collection; therefore, herbarium data were not included when mapping kelp records along the coastline through time. Site records (coordinates) for *L. hyperborea* were downloaded or donated from the Global Biodiversity Information Facility (10 January 2020, www.gbif.org), Ocean Biogeographic Information System (10 January 2020, www.obis.org), National Biodiversity Data Centre (19 September 2019, www.biodiversityireland.ie), and the Environmental Protection Agency (17 September 2019). All data were concatenated and quality‐filtered for duplicate records (i.e., same coordinates, same date) and correct geographical location (i.e., points on land were removed). Data were then sorted by year of recording and number of years recorded to highlight sites accessed earliest within the country (“period of first record”; Figure 1) and of great interest (“number of years recorded”; Figure 2). Overall, recording effort increased as we approach the present day, with a boom in the 1990s, however, few sites were recorded multiple times (Table 2).

**TABLE 2:** The total number of records for *Laminaria hyperborea*grouped by time period from pre‐1950 to 2018 (present day)

	<1950	1950–69	1970–89	1990–99	2000–09	2010–18	Total
1	0	6	344	558	293	495	1696
2	1	3	30	524	26	54	638
3	0	4	5	5	2	4	20
4	0	3	3	0	0	0	6
5	0	1	0	0	0	0	1
6	0	3	0	0	0	0	3
Total	1	20	382	1,087	321	553	2,364

**FIGURE 3:**
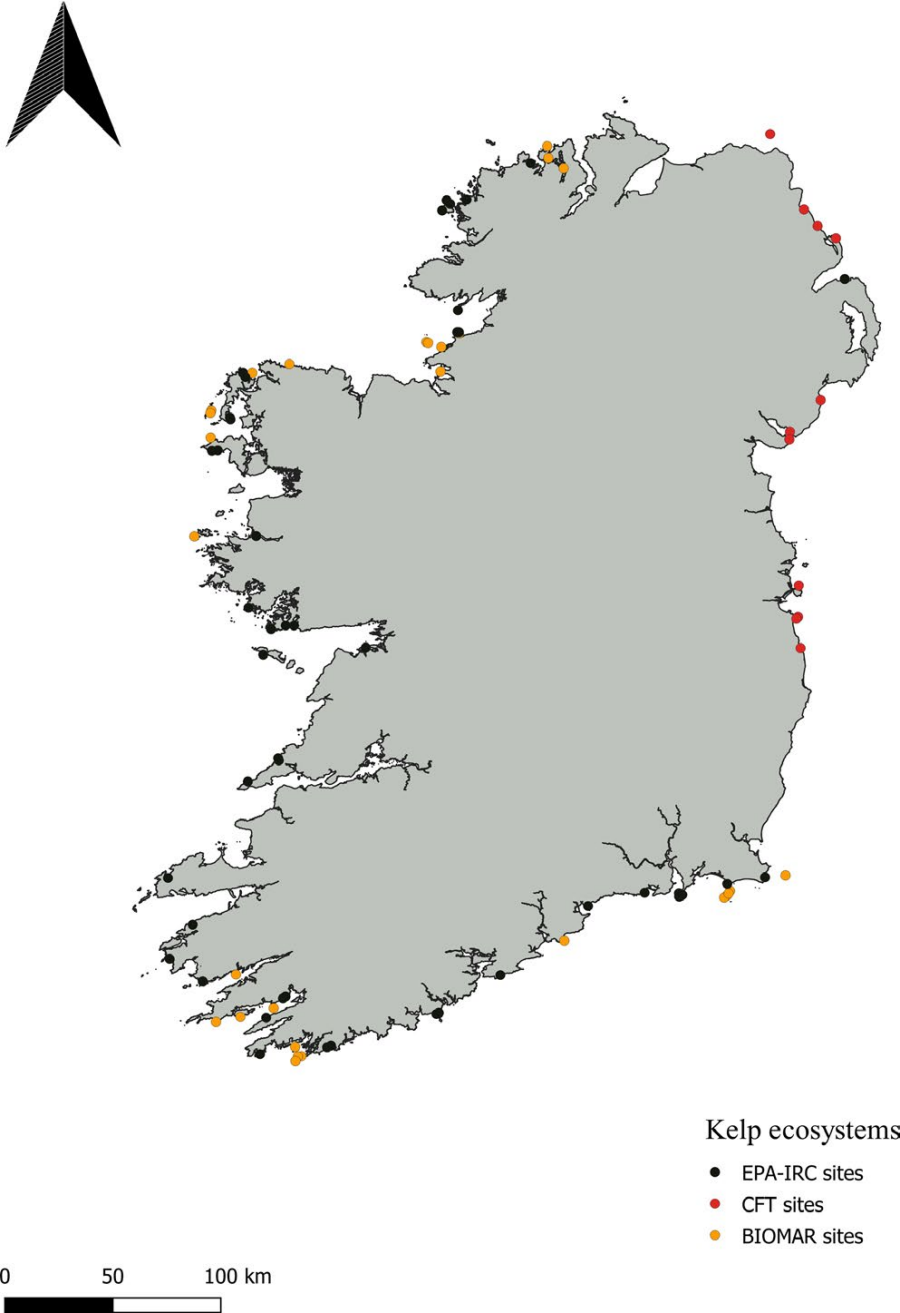
Records of kelp forest ecosystems from the BIOMAR study (Picton & Morrow, 2006), IRC and EPA research programs (Schoenrock et al., 2020), and Comhairle Fo‐Thuinn (CFT) dive clubs around Ireland. Many locations overlap with locations from database queries (Figures 1 and 2), indicating that GBIF and OBIS records could often indicate presence of kelp forests

Records for kelp forest sites were provided through recreational Comhairle Fo‐Thuinn (CFT) dive clubs throughout Ireland, the BIOMAR data set (Picton & Morrow, 2006), and recent Irish Research Council and Environmental Protection Agency projects (Schoenrock et al., 2020). These were analyzed separately to highlight the distribution of kelp ecosystems (not just individual kelp sightings or drift algae). Kelp ecosystems (including survey data) have significantly fewer records (Figure 3) in comparison with *L. hyperborea* records (Figure 1); however, there is some overlap. We hope that future observations of *L. hyperborea* will include more metadata, such as whether the kelp was found *in situ* *,* and whether it was in a kelp forest, park, other habitat, or on a strandline (as beach wrack). The difference between a kelp forest or park is generally described as a reduced density of large kelp individuals, generally adjacent to a kelp forest, although there is no ecological distinction to date (Parr, 2020).

## 3 HISTORICAL RECORDS, 1700–1900

**FIGURE 4:**
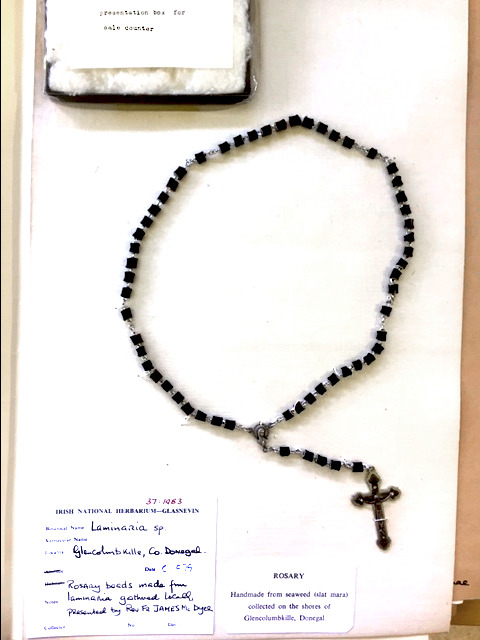
Image of a kelp rosary within the herbarium at The National Botanic Gardens of Ireland. The “beads” of the rosary are likely made from made from the stipes of *Laminaria digitata* collected from Glencolumbkille, County Donegal

During the 1700s, many natural historians began extensive descriptions of what were termed “the Algae” from the initial growth to fructification (formation of reproductive structures) in intertidal species (Greville, 1830 and authors listed within). Some of these researchers focused on seaweeds of the British Isles (present‐day Éire and the United Kingdom), and many were women and clergymen (e.g., Reverend David Landsborough of Glasgow, 1847) who had a keen interest in the natural world (see Table 3). This work may have been driven by the need to understand marine harvest because “kelp,” potash of all seaweeds, was an economic resource used in agriculture, as packaging material, and an iodine source (Harvey, 1849), and this is certainly a driver for renewed interest in seaweeds from the 1940s (Ara Mara; South & Titley, 1986) to today (Monagail & Morrison, 2020). Cultural uses of kelp were also observed: One herbarium record from The National Botanic Gardens of Ireland is a rosary made from the stipes of *L. digitata* collected from Glencolumbkille, County Donegal (Figure 4). Historical records of Irish or Irish‐based phycologists is very thoroughly outlined in Guiry (2012); but unfortunately, the information provided at this time does not define the distribution of any recorded seaweeds, but instead refers to ecological aspects like zonation on the shoreline (e.g., the taxon now termed *S. latissima* was thought to only live between high and low tides). *Laminaria* spp. and other seaweeds were often described as tangles (Harvey, 1849), and herbarium records in The National Botanic Gardens of Ireland and Trinity College indicated many *Laminaria* spp. could easily be grouped into what was *Laminaria phyllis* (present‐day *L. digitata*) which Harvey noted as having multiple ecotypes, some of which are actually *L. hyperborea* (Harvey, 1841). Previous names (not misnomers) for *L. hyperborea* include *Fucus hyperboreus* Gunnerus, *Fucus scoparius* Strom 1762, *Hafgygia cloustonii* (Edmondston) Areschoug, 1883, *Laminaria cloustoni* Edmondston, *Laminaria hyperborea* f. *compressa* Foslie, 1884 (Guiry & Guiry, 2020).

**TABLE 3:** A list of phycologists and natural historians who worked on seaweed communities in the UK and Ireland from the 1700s to 2010, enabling our understanding of these communities

Name	Year	Record
John Templeton	1766–1825	Flora Hibernica, Ulster Museum
Dawson Turner	1775–1858	British Phycologist
James Lawson Drummond	1783–1853	Irish Phycologist
Ellen Hutchins	1785–1815	Irish Phycologist
George Crawford Hyndman	1796–1867	Irish Phycologist
William Thompson	1805–1852	XXII. Additions to the fauna of Ireland
Anne Elizabeth Ball	1808–1872	Irish Phycologist
William Henry Harvey	1811–1866	Irish and British Phycologist
William McCalla	1814–1849	Naturalist, Roundstone County Galway
J. Cocks Esq., M.D.	1859	Observations on the growth and time of appearance of some of the marine algae, &c.
Alexander Stewart & Corry	1858	Flora of the north‐east of Ireland
Robert L. Praeger and Arthur Disbrowe Cotton	1865–1953	Irish naturalist, Clare Island surveys
Captain Cary (Christian name unknown)	1869–1912	Sea Captain, Four marine algae herbaria to National Botanical Gardens
S. O. Gray	1867	British seaweeds: an introduction to the study of the marine algae of Great Britain, Ireland and the Channel Islands
E. M. Holmes	1883	New British Marine Algae, A revised list of the British marine algae, Appendix
E. A. L Batters	1895	New or critical British marine algae, On some new British marine algae, A revised list of the British marine algae, Appendix
Henry Hanna	1898–1899	Irish Phycologist
T. K. Rees	1935	The marine algae of Lough Ine
M. J. Lynn	1937	Notes on the algae of the district of Whiterock, Strangford Lough
Agnes T. Brennan	1945	Notes on the Distribution of Certain Marine Algae on the West Coast of Ireland
W. A. P. Black	1950	The seasonal variation in weight and chemical composition fo the British Laminariaceae
M. Parke	From 1950	British Phycologist
H. M. Parkes	1958	A general survey of the marine algae of Mulroy Bay, County Donegal, A list of marine algae from the Wexford Coast
Peter S. Dixon	From 1960	British Phycologist
L. H. Colinvaux	1965	A partially annotated bibliography of the algae of Ireland
Joanna M. Kain (Jones)	1930–2017	British Phycologist
Trevor A. Norton	From 1967	Irish Phycologist
M. J. P Scannell	1969	Unpublished Records of marine algae made mainly in the County Waterford by Thomas Johnson and Matilda Knowles
Michael D. Guiry	From 1969	Irish Phycologist
Colin Pybus	1975	Some notes and observations on encrusting red algae from County Galway
Osborne Morton	1978	Interesting records of algae from Ireland
J. P. Cullinane	From 1966	Marine Algal Records from the South Coast of Ireland
Patrick M. Whelan	From 1976	Marine Algal Records from the South Coast of Ireland
Mairin de Valera	From 1939	Littoral and benthic Investigations of the west coast of Ireland, X. Marine algae of the northern shores of the Burren, County Clare
Matthew J. Dring	From 1980	British Phycologist
Linda Mary Irvine	From 1969	British Phycologist
Cilian Roden	1981	Noteworthy marine algae from County Dublin
Christine A. Maggs	From 1983	Irish and British phycologist
Barry Egan	1983	Notes on the marine algae of Ballycotton Bay, County Cork
Sue Hiscock	From 1982	British Phycologist
Juliet Brodie	From 1988	British Phycologist
Dagmar B. Stengel	From 1994	Irish Phycologist
Sammy De Grave	From 2000	Irish maerl beds
Cynthia Trowbridge	From 2001	Invasive algal species in Ireland
Stephan Kraan	From 2000	Irish Phycologist
Fabio Rindi	From 1994	Molecular species identification
Liam Morrison	From 2004	Irish Phycologist

Note

People are listed with known names or initials, dates of activity specifying life span, date of only phycology publication or first year of publication if still active (noted as “From…”), and specific information about the phycologist or naturalist (publication information or occupation).

Marine algal communities in the British Isles were originally described as dominated by “Olive series” (Phaeophyta), with many red algae (3/8 of species) and greens (1/4 species: Harvey, 1849). Interesting notes on the ecology of seaweeds include seaweed support of food webs and community structure: “The Algae, therefore by supporting the base, support the structure” (Harvey, 1849), which is potentially the first description of seaweeds as ecosystem engineers and/or providing ecosystem services. Observations were also noted on ecological interactions between coralline algae and fleshy algae (Lamouroux, 1826), the annual phenology (annual growth patterns) of marine algae, variation in zonation from subtidal to intertidal (“land flora”) across regions, and the distribution of dominant brown algae (Cocks, 1859; Harvey, 1849). Most of these studies were restricted to coastlines where seaweed could be easily observed. Cocks (1859) even notes his thought that there would be little space for seaweed below the tidelines (i.e., subtidal). There could be a good deal of data on subtidal marine algae in sounding records of the British Admiralty dating back to 1,580 which would have refuted this idea; however, these records were and are not easily accessible. Up to the twentieth century, much of phycology in Ireland was focused on species descriptions and distributions to the extent of providing “presence” data on certain portions of the coastlines (broadly noted as “Northern Ireland” or “County Cork” for example) which is still an issue with some reports today (see Scally, Pfieffer, & Hewitt, 2020).

## 4 HISTORICAL RECORDS, 1900–2018

Some of the first comprehensive surveys of natural environments occurred on Lambay and Clare Island in the early 1900s, and these multidisciplinary reports provide a comparison of intertidal algal communities between 1910 to the 1990s (Cotton, 1909, 1912), but more recent surveys have collected subtidally, expanding the flora record for these locations (Rindi & Guiry, 2004). Subtidal observations became easier for phycologists with the advent of diving bells and, later, the self‐contained underwater breathing apparatus (SCUBA). Jack Kitching first described methods for studying sublittoral ecology in the UK using a diving helmet in the 1930s, which subsequently led to the first observation of the species associations in *Laminaria* spp. forests, including the dominance of *L. hyperborea* (formerly *L. cloustoni*; Kitching, Machan, & Gilson, 1934). He later brought that equipment to Ireland where he intensively studied the ecology of Lough Hyne (or Ine) with generations of students, providing the basis for kelp forest ecology in this region of the world which would later be proliferated by Joanna Kain (Jones) using SCUBA from 1960–late 1980. The seaweeds of Lough Hyne were first described by Rees (1931), later followed up by Maggs,Freamhainn, & Guiry (1983) who also contributed to many reports on the biotope “kelp forests” in Ireland and the UK (Birkett et al., 1998; C.A. Maggs, M.D. Guiry, & M.J. Dring, unpublished data). Kain's work defined *L. hyperborea's* population dynamics (Kain, 1963), reproduction (Kain & Jones, 1964), competition and growth (Creed, Kain, & Norton, 1998; Kain, 1962, 1969, 1976a, 1977), and description of succession and subcanopy/understory seaweeds (Kain, 1976b, 1982, 1989). This research, alongside that of Norwegian and French phycologists, forms the basis of our understanding of kelp forest ecology in Ireland (summarized in Kelly, 2005), though more recent research projects in the UK and Ireland aim to supplement this knowledge base with modern data (e.g., Burrows et al., 2014; Schoenrock et al., 2020).

The first distribution record with multiple georeferenced data points of large seaweeds in the UK and Ireland was published by Crisp and Southward in 1958, as a side note to their record of intertidal invertebrates (Crisp & Southward, 1958). From 1950 to 1990, multiple studies referenced seaweeds in specific regions (see Table 3); for instance, Morton (1994) noted the abundance of marine algae in Northern Ireland by county. The BIOMAR survey (Picton & Morrow, 2006) of marine habitats across Ireland summarized species associated with subtidal kelp forest habitats in the 1990s using SACFOR abundance scales for taxa (super abundant, abundant, common, frequent, occasional, and rare) that could be repeated over time in the same locations. This was followed by a repeat survey of regions in Crisp and Southward (1958) by Simkanin et al. (2005) which highlighted increases or declines of species abundance over the 45 years between studies. Declines in northerly species in these intertidal habitats occurred in five of 12 species (including *L. hyperborea* and *S. latissima*), while increases occurred in one of 12 species (e.g. the invasive barnacle *Australminius modestus*; Simkanin et al., 2005). In contrast, one of nine southerly species declined in abundance, despite the trend in Europe for southerly species to expand their northern ranges (e.g., *L. ochroleuca*: Schoenrock, O'Callaghan, O'Callaghan, & Krueger‐Hadfield, 2019; Smale, Wernberg, Yunnie, & Vance, 2015). Merder et al. (2018) later showed that community similarity indices in Simkanin et al. (2005)'s data were more influenced by the environmental variables wave energy and Chl *a* concentration than sea or air temperature, which resulted in differences in communities from east to west coasts. The formation of Seasearch Ireland in 2009 has boosted records of subtidal habitats, to the scale that most recent *L. hyperborea* records in Ireland are supplied by citizen scientists (2010–2018, Figure 1). The remaining data are from research agencies like the Environmental Protection Agency or National Parks and Wildlife Services.

BIOMAR data are unique in the fact that they can be analyzed to highlight the impact that kelp species and region have on faunal assemblages within kelp ecosystems (Tables 4 and 5). SACFOR scales were given a numerical value (0 = absent, 1 = rare, 2 = occasional, 4 = frequent, 5 = common, 6 = abundant, and 7 = super abundant) for each site record, and a Bray–Curtis similarity matrix was created with species data across sites, and finally, similarity of species compositions within kelp forests, (a) within the same geographical region (Table 4) and (b) within forests dominated by different kelp species (Table 5), were evaluated using an analysis of similarity (ANOSIM; Clarke & Gorley, 2006). Regional differences were apparent in kelp communities; for example, more species contribute to community similarity in kelp forests in west Ireland than in other regions (Table 4). Dominant kelp species also affected community assemblages, but too few replicates exist in mixed and *A. esculenta* forests to define species driving differences (Table 5). When compared with a recent study in the west of Ireland (Table 1), species associated with *L. hyperborea* forests are notably different (Table 5), potentially due to the quantitative versus qualitative data collection methodology, and survey focus. For instance, kelp blades where many hydroids reside (e.g., *Electra pilosa*, Tables 4 and 5) were not included in the swath surveys used for community analysis in Schoenrock et al. (in review). Moving forward, creating a standard monitoring methodology would benefit analysis of data and highlight [changing] patterns in species distribution and habitat usage over time.

**TABLE 4:** ANOSIM analysis of BIOMAR abundance scales for 31 kelp forest sites in five regions: southwest (County Cork), southeast (County Wicklow and Wexford), west (County Kerry, Clare, Galway and Mayo), northwest (County Sligo and Donegal), and north (north County Donegal)

Geographic region	Species	% Contribution
Southwest 6.99% similarity S = 31.78 *N* = 9	*Callopora lineata*	51.1
	*Crisia denticulata*	14.6
	*Alcyonidium diaphanum*	10.57
	*Electra pilosa*	7.13
	*Scrupocellaria* spp.	7.13
Southeast 19.38% similarity S = 78 *N* = 6	*Flustra foliacea*	32.98
	*Electra pilosa*	29.79
	*Membranipora membranacea*	25.04
	*Chaetopterus variopedatus*	5.03
West 11% similarity S = 41.15 *N* = 7	*Electra pilosa*	24.7
	*Callopora lineata*	16.72
	*Flustra foliacea*	10.47
	*Crisia eburnea*	7.97
	*Bicellariella ciliata*	5.96
	*Parasmittina trispinosa*	5.96
	*Chaetopterus variopedatus*	5.9
	*Phyllodoce laminosa*	4.47
	(O) *Spirorbidae*	4.47
	(O) *Terebellidae*	4.47
Northwest 0% similarity S = 50.67 *N* = 6	No data	
North 57.07% similarity S = 44 *N* = 3	*Electra pilosa*	45.13
	*Crisia denticulata*	15.93
	*Scrupocellaria*	15.58
	*Crisia eburnea*	11.68
	*Flustra foliacea*	11.68

Note

Similarity of communities in regions, species richness (S), and site number (*N*) are displayed along with species driving similarity and % contribution.

**TABLE 5:** ANOSIM analysis of BIOMAR abundance scales for 31 kelp forests composed predominantly of *Laminaria hyperborea* but three sites dominated by *Alaria esculenta* and 1 mixed kelp forest (*L. hyperborea* and *Saccharina latissima*)

Kelp species	Species	% Contribution
*L. hyperborea* 13.85% similarity S = 51.74 *N* = 27	*Electra pilosa*	26.68
	*Flustra foliacea*	16.51
	*Callopora lineata*	15.44
	*Membranipora membranacea*	9.35
	*Crisia denticulata*	7.17
	*Scrupocellaria* spp.	5.91
	*Crisia eburnea*	4.14
	*Chaetopterus variopedatus*	2.8
	*Alcyonidium diaphanum*	2.66
*L. hyperborea* and *S. latissima* S = 48 *N* = 1	No data	
*A. esculenta* S = 11 *N* = 3	No data	

Note

S is species‐richness, and *N* is number of sites, and % contribution of fauna driving similarity within kelp forests.

In summary, distribution records for kelp have fluctuated over time in terms of recording effort and regions visited. The focus of study has progressed from basic species description and use as a resource from the 1700s–1910s, expanding to disciplines like ecology, evolution, and natural product chemistry which are facilitated by technology (Young et al., 2015). Present‐day investigations utilize species distribution models to project future distributions of seaweeds based on the habitat suitability or environmental forcing associated with records of species presence. Yesson, Bush, Davies, Maggs, and Brodie (2015a) modeled the distribution of kelp and fucoid species in the UK and Ireland using data from herbaria and online databases and found (a) most distribution data comes from studies after 1970 (in contrast to the present review where the majority were post‐1990) and (b) different environmental requirements for each species. Non‐natives, like *U. pinnatifida,* are found in areas with high average temperatures (but also restricted to man‐made or modified structures, e.g., harbors), while the native *A. esculenta* is found in regions with colder average temperatures (Yesson et al., 2015a) and is thought to be more susceptible to temperature than the *Laminaria* spp. of the region (Müller, Laepple, Bartsch, & Wiencke, 2009). *Laminaria* spp. are influenced more by substrate type than temperature or light in current distributions, and *L. hyperborea* is thought to cover 48, 654 km^2^ of coastline in the UK and Ireland, specifically on rocky substrate with moderate wave exposure (Yesson et al., 2015a). Species distribution models indicate that *L. hyperborea* blankets all coastlines in Ireland that are not adjacent to major freshwater sources (see figure 5 in Yesson et al., 2015a), though this potentially overestimates its distribution along the east coast because the coastline has more sand/mud/marsh habitats than rocky coastline (Neilson & Costello, 1999). More interestingly, the study also indicates regions suitable for species range expansions including Bellmullet County Mayo, where the first record of *L. ochroleuca* in Ireland was noted in 2018 (Schoenrock, O'Callaghan, O'Callaghan, & Krueger‐Hadfield, 2019). Models that factor in climate change predictions show kelps retracting northward (Assis et al., 2016), and this has already been noted in species found in Ireland (Simkanin et al., 2005; Yesson, Bush, Davies, Maggs, & Brodie, 2015b). These findings indicate the need for better habitat mapping tools, which are superior to point records, but also difficult to achieve with species in the sublittoral where remote sensing and monitoring require significant investment of resources.

## 5 RESILIENCE AND MONITORING OF *LAMINARIA HYPERBOREA* COMMUNITIES

Recovery of kelp ecosystems after large disturbances is an important aspect of resilience in the marine environment. *Laminaria hyperborea* is a long‐lived species, reaching ~15 years of age in west Ireland (maximum of 18 years in Finnmark, Norway; Sjøtun, Fredriksen, Lein, Rueness, & Sivertsen, 1993) but with an average age of 4 years, where juvenile kelps reappear throughout the year as canopy is removed by storms, regenerating the populations annually (Schoenrock et al., personal communication). There is no destructive grazing in *L. hyperborea* communities in this region. The common urchin, *Echinus esculentus,* does not destructively graze gametophytes or juvenile sporophytes in the subcanopy, and generally, adult kelps are left untouched (Sjotun, Christie, & Fossa, 2006). Another urchin species (*Paracentrotus lividus*) was overfished in the twentieth century (Barnes & Crook, 2001), and small populations of the green urchin (*Strongylocentrus droebachiensis*) do not pose a threat in Ireland as they do in Norway and urchin populations generally do in other regions of the world (Estes & Duggins, 1995; Hagen, 1995; Ling et al., 2015). The blades of *L. hyperborea* annually regenerate, starting growth in winter and reaching maximum length mid‐summer, and producing sori from October to March (Kain & Jones, 1964). The zoospores produced within sori disperse ~200 m and settle to develop into gametophytes and following fertilization, juvenile sporophytes (Fredriksen, Sjøtun, Lein, & Rueness, 1995). This life cycle may facilitate resilience of kelp populations, allowing for refuge from environmental and biological stressors as either (a) a large sporophyte is too large for grazers or (b) a microscopic stage is safe from storms or otherwise that would uproot large sporophytes (i.e., bet‐hedging: Lubchenco & Cubit, 1980). However, our understanding of the role of kelp gametophytes as a spore bank is limited to only a handful of studies (e.g., Robuchon, Couceiro, et al., 2014).

Resilience may also be conferred through genetic diversity as genetic variation is the essential evolutionary mechanism with which species can respond to environmental stochasticity. Larger, outcrossed populations tend to be more genetically diverse, than smaller, often inbred, populations. Studying these patterns in the sea can be challenging as not all predictions from terrestrial environments necessarily apply (i.e., chaotic genetic patchiness: Galindo, Olson, & Palumbi, 2006; Selkoe et al., 2010). Population genetic tools provide a powerful way with which to study how genetic diversity is partitioned in natural populations, and by extension, patterns of connectivity and population structure in the sea (seaweed population genetics reviewed in Krueger‐Hadfield & Hoban, 2016; Valero et al., 2011). Myriam Valero et al. (2011) reviewed the current state of the literature on population genetic patterns in kelp, with most of the studies centered on kelps in Europe (mainly in France [Brittany] and Portugal), Australia, Chile, and California. Interestingly, species that had population genetic data (*Macrocystis pyrifera*, *Lessonia nigrescens*, and *L. digitata*) harbored the highest levels of diversity in areas with strong harvesting pressure. Population connectivity (with kelp species) is largely affected by habitat discontinuity (e.g., Billot, Engel, Rousvoal, Kloareg, & Valero, 2003), and patterns of isolation by distance are common (e.g., Robuchon, Le Gall, Mauger, & Valero, 2014). Understanding how genetic diversity is partitioned and how populations are connected to one another is a necessity in order to determine how populations could recover from harvesting (Robuchon, Le Gall, et al., 2014) or from disturbances, such as heatwaves seen within the Pacific Ocean (Wernberg et al., 2019).

Myriam Valero et al. (2011) conclude that “while kelps are economically and ecologically important, only a few studies have attempted to assess genetic variation within kelp populations and on small scales.” Likewise, few studies have included temporal scales in monitoring efforts for genetic diversity. This is even more apparent along the coast of Ireland where until recently there were no systematic studies of the population genetics of kelp species. Schoenrock, O'Callaghan, O'Callaghan, & Krueger‐Hadfield, (2019) found that genetic diversity in the non‐native *L. ochroleuca* was comparable to the southern range edge of this species rather than closer populations in France. Moreover, the excess of heterozygotes at Scots Port in Bellmullet was interpreted as the result of recent admixture following a founder event (Schoenrock, O'Callaghan, O'Callaghan, & Krueger‐Hadfield, 2019). In addition, glacial refugia, or areas of long‐term persistence during glacial maxima, have been predicted for *L. hyperborea* along the southern coastline of Ireland (Assis et al., 2016), suggesting these areas may harbor unique genetic diversity. Schoenrock et al. (2020) confirmed that the highest levels of allelic diversity and heterozygosity were found in the *L. hyperborea* population at Lough Hyne in the southwest of Ireland. They genotyped seven other populations along the west coast from County Cork to County Donegal and found patterns of decreasing diversity as well as isolation by distance. However, only eight sites spread over much of the west coast of Ireland were included in this study, rendering it difficult to study smaller scale patterns in genetic structure. Ongoing analysis of forty‐two sites along the entire coastline of Ireland should help investigate this further (Schoenrock et al., personal communication), with temporal sampling to provide insight into the genetic stability of *L. hyperborea* in Ireland (*sensu* Valero et al., 2011). Continued monitoring of these genetic resources, as well as expanding the number of taxa included (other canopy species like *S. polyschides* or *S. latissima*), will be important moving forward.

Monitoring kelp forest habitats in Ireland is a difficult task, as the reticulated coastline is highly exposed to the dynamic North Atlantic Ocean. A recent survey indicates that healthy kelp ecosystems can be quantified through density and height of the kelp bed using single beam sonar with video validation of species ID (Biosonics; Scally et al., 2020). This technology is incredibly helpful when creating a mapping tool for subsurface forests; however, population surveys from the west of Ireland indicate that density and height of stable kelp forests have huge fluctuations throughout the year (peak in summer) with an average of 20.21 individuals/m^2^, few of which are canopy forming; greatest kelp height is observed in shallow habitats (~2 m depth, LAT; Schoenrock et al., 2020), although forests reach ~15 m depth on islands off Ireland's coasts (C.A. Maggs, M.D. Guiry, & M.J. Dring, unpublished data). A better monitoring scheme should be put in place and could include the use of sonar (see Blight et al., 2011; Mac Craith & Hardy, 2015) or satellite platforms to map these ecosystems. Although more typically used to monitor blooms in estuarine and coastal habitats (*Ulva* spp.: Mora‐soto et al., 2020), satellite data are also useful in mapping kelp species that span the water column and were pioneered in the east Pacific (*M. pyrifera:* Mora‐soto et al., 2020; Cavanaugh, Siegel, Kinlan, & Reed, 2010). Simms and Dubois (2010) created a method for submerged kelp beds in the northwestern Atlantic, which could potentially be used on the subtidal *L. hyperborea* forests in Ireland.

A recent review by Duffy et al. (2019) classifies marine macroalgae and seagrass monitoring as an “emerging priority” globally for ocean and coastal management. Tiered observation systems are proposed to monitor broadscale patterns at wider intervals, using remote‐sensing coupled with underwater observations, but detailed *in situ* sampling annually at selected sites is also advised, to capture information such as taxonomic associations to bolster data and understanding of ecosystem function (Duffy et al., 2019). Ireland and other small countries are unlikely to devote substantial resources to regular kelp forest monitoring without more apparent delivery of ecosystem services. A foreseeable way to monitor status and trends in these habitats would be to ground‐truth remote sensing technology and supplement this effort with citizen scientist observations. Seasearch Ireland provides a scheme for CFT divers to “adopt a site” and kelp forests could be targeted in their region, documenting the habitats kelp species are found in or form. The presence of associated faunal species abundance, particularly large mobile species that are easier to see (see indicator species in Table 1), would help to create a data set where fluctuations in species assemblages within kelp forests could be monitored, filling key information gaps on the ecosystem services provided by these ecosystems (e.g., Bertocci, Araújo, Oliveira, & Sousa‐Pinto, 2015). National governments should be committed to monitor kelp ecosystems under European Union (EU) environmental legislation (EU Marine Strategy Framework Directive, MSFD) (European Commission, 2008), and the EU Water Framework Directive (European Commission, 2000) because ecosystem‐based management (EBM) is central to the legislations objectives (Berg, Fürhaupter, Teixeira, Uusitalo, & Zampoukas, 2015), including healthy commercial fish and shellfish stocks (MSFD descriptor 3) and healthy marine food webs (MSFD descriptor 4).

## 6 CONCLUSIONS

Studies of kelp forests in Ireland are historically rare and contain mostly qualitative information. Kelp records with georeferenced data points date back to 1913 and continued over the decades, with a pulse in records from the 1990s onward. Most records are single sightings of *L. hyperborea*, indicating that either people do not record multiple sightings of the same kelp forest, or many regions are not revisited. Recording effort should move toward documenting kelp ecosystems (presence of a forest) as well as abundance of “indicator species” within using standardized methodology. This would boost evidence that kelp forests are indicators of good environmental status and could be used operationalize MSFD legislation. Maintaining resilience of kelp forests and their associated species is important not only for the ecosystems, but the services they provide to civilization, which can be achieved through monitoring habitats and management of stressors (Krumhansl et al., 2016). Development of a remote sensing mapping tool (via satellite or otherwise) would aid in monitoring the distribution kelp forest distributions.

## CONFLICT OF INTEREST

All authors declare no conflict of interest.

## AUTHOR CONTRIBUTION

**Kathryn Schoenrock:** Conceptualization (lead); Data curation (lead); Formal analysis (lead); Funding acquisition (lead); Investigation (lead); Methodology (lead); Project administration (lead); Resources (lead); Validation (lead); Visualization (equal); Writing‐original draft (lead); Writing‐review & editing (equal). **Kenan M. Chan:** Visualization (supporting); Writing‐review & editing (supporting). **Tony O'Callaghan:** Data curation (equal); Resources (supporting); Writing‐review & editing (supporting). **Rory O'Callaghan:** Data curation (supporting); Resources (supporting); Writing‐review & editing (supporting). **Aaron Golden:** Methodology (supporting); Writing‐original draft (supporting); Writing‐review & editing (supporting). **Stacy A. Krueger‐Hadfield:** Resources (supporting); Writing‐original draft (equal); Writing‐review & editing (supporting). **Anne Marie Power:** Data curation (supporting); Project administration (supporting); Writing‐original draft (supporting); Writing‐review & editing (supporting).

## Data Availability

All data used within this manuscript are freely available in National Herbaria and online databases: www.obis.org, www.gbif.org, www.biodiversityireland.ie, and http://erc.epa.ie/droplet/.
